# Exploring the potential benefits of clonidine for anxiety disorders

**DOI:** 10.1017/S1092852925100266

**Published:** 2025-05-16

**Authors:** Esha Aneja B.S., Soojae Hollowell, Thomas Schwartz

**Affiliations:** 1 Department of Medicine, California Northstate University, Elk Grove, CA, USA; 2 Department of Psychiatry, SUNY Upstate Medical University, Syracuse, NY, USA

**Keywords:** Anxiety disorders, clonidine, alpha-2 agonist, anxiolytic, generalized anxiety disorder

## Abstract

Anxiety disorders, characterized by excessive fear and behavioral disturbances, are among the most prevalent psychiatric conditions, yet treatment options remain suboptimal for many patients. Clonidine, an alpha-2 adrenergic receptor agonist, has shown potential anxiolytic effects and may address treatment-resistant cases. This review explores the efficacy, safety, and mechanism of clonidine as a pharmacological option for anxiety disorders, with emphasis on its role in modulating noradrenergic dysfunction and its potential synergistic effects with existing therapies. A literature review was conducted to evaluate clinical studies, case reports, and comparative trials on clonidine’s use in anxiety disorders, focusing on its pharmacological profile, efficacy, and tolerability. Evidence suggests clonidine may reduce anxiety symptoms, particularly in treatment-resistant cases and specific populations, such as pediatric patients and those with comorbid psychiatric disorders. Its mechanism involves modulating norepinephrine release and glutamatergic pathways. Case studies and small trials highlight its potential in reducing cognitive symptoms of anxiety, but inconsistencies in efficacy and side effects, including sedation and hypotension, were noted. Comparative studies suggest clonidine may have similar efficacy to SSRIs in some cases but lack large-scale validation. Clonidine presents as a promising pharmacotherapeutic option for anxiety disorders, particularly in cases resistant to conventional treatments or in patients with contraindications to other typical medications. Its mechanism of action, tolerability, and potential synergistic effects with existing therapies underscore the need for continued exploration and clinical trials to establish its optimal role in anxiety disorder management.

## Introduction

Anxiety disorders, including generalized anxiety disorder (GAD), social anxiety disorder (SAD), and specific phobia (SP), are among the most common psychiatric conditions, with a lifetime prevalence of approximately 33% in the United States.^1^ They are pervasive mental health conditions with varying onset and course patterns across different age groups.^2^^,^ ^3^ While these disorders often start in childhood, adolescence, or early adulthood, they tend to peak in middle age before showing a decline in older age.^1^ Research suggests that anxiety disorders can be chronic, lasting for years or even decades, but this does not imply lifelong persistence for all individuals affected.

The National Comorbidity Survey Replication (NCS-R) provides valuable insights into the prevalence and age-related distribution of anxiety disorders. Among adolescents aged 13 to 17 years, anxiety disorders emerge as the most common class of mental disorders, with SPs and SAD being particularly prevalent.^1^ Interestingly, the lifetime prevalence rates for certain anxiety disorders, such as panic disorder (PD), GAD, and SAD, are lower in adolescents compared to adults aged from 18 to 64 years.^1^

The World Health Organization reports a significant increase in the global prevalence of anxiety disorders, necessitating a focused approach toward developing novel treatment strategies. Despite the high prevalence and substantial impact on individuals’ daily functioning, recent research on innovative medication treatments for anxiety disorders has been relatively limited compared to other psychiatric disorders.

GAD is a common mental health condition characterized by excessive worry and anxiety across various domains of life. Current treatments primarily involve selective serotonin reuptake inhibitors (SSRIs), serotonin-norepinephrine reuptake inhibitors (SNRIs), benzodiazepines (BZs), and other medications targeting various neurotransmitter systems. The first-line treatments for PD, GAD, and SAD are SSRIs and SNRIs.^2^ While SSRI antidepressants are frequently prescribed for GAD, medications targeting noradrenergic pathways (SNRI) have also demonstrated efficacy in managing anxiety symptoms.^1^^,^^4^^,^^5^

## Anxiety disorders treatments

Clonidine, a norepinephrine-manipulating drug, is primarily known for its antihypertensive properties. It has gained some attention for its anxiolytic effects. However, there exist few studies that have explored clonidine’s efficacy in anxiety disorders. Clonidine has been used experimentally to modulate anxiety and dampen noradrenergic function and has shown clinical utility in conditions such as opiate withdrawal and rapid eye movement (REM) arousal disorders.^6^^,^^7^ Clonidine sees fair use in childhood attention deficit hyperactivity disorder (ADHD) and treatment when stimulants create agitation or insomnia. It is often used off-label for anxiety disorder. It is typically used when BZs are felt too risky. Clinically, there have been no recent clinical studies or drug trials of clonidine use in PD, SAD, and GAD.^8^ Given its pharmacological profile and effects on physiological parameters, clonidine represents a potential candidate for investigating the role of noradrenergic dysfunction associated with anxiety disorders. The precise role of noradrenergic activity in GAD, for example, remains unclear, prompting investigations into novel therapeutic approaches. Despite the availability of Food and Drug Administration (FDA)-approved medications and psychotherapies, a considerable number of patients do not achieve satisfactory results with existing treatments. Because anxiety disorders are prevalent psychiatric conditions with considerable impacts on individuals’ quality of life and societal costs, there exists a need for alternative pharmacotherapies to address the challenges that are associated with managing anxiety disorders effectively.

## Current treatments for anxiety disorders

Currently available treatments for anxiety disorders include selective SSRIs, SNRIs, BZs, and cognitive behavioral therapy (CBT). SSRIs and SNRIs are considered first-line pharmacologic options, but many patients experience incomplete relief or intolerable side effects.^1^ BZs, while effective, carry risks of dependence and cognitive impairment.^9^ Other treatment options include beta-blockers, buspirone, and certain anticonvulsants, but their efficacy is variable.^10^ Given these challenges, clonidine’s unique mechanism of modulating noradrenergic activity makes it an intriguing candidate for anxiety treatment.

## Material and Methods

This review was conducted as a narrative literature review rather than a systematic review. A comprehensive search of PubMed, PsycINFO, and Embase was performed using the key terms: “clonidine,” “anxiety disorders,” “noradrenergic dysfunction,” and “alpha-2 adrenergic agonists.” Studies published from 1980 to 2024 were included. While systematic reviews provide higher levels of evidence, a narrative review format was chosen due to the limited number of randomized controlled trials (RCTs) on clonidine for anxiety disorders. This limitation is acknowledged in the discussion section.

## Mechanism of action

Clonidine’s mechanism of action involves binding to presynaptic alpha-2 adrenergic receptors, leading to reduced noradrenaline release and subsequent downward modulation of sympathetic nervous system activity.^11^ This allows blood vessel relaxation and lowers blood pressure. When these receptors are activated, there is a reduction in the release of excitatory neurotransmitters, including norepinephrine by reducing calcium into presynaptic neurons. Through modulating norepinephrine, alpha-2 agonists can decrease the excitability of glutamate neurons and lower their transmission as well. By changing glutamatergic input from the cortex into the midbrain, there may be beneficial changes in downstream monoamine activity. The limbic system, including the amygdala and hippocampus, is rich in adrenergic receptors. By inhibiting the release of norepinephrine in the limbic system, alpha-2 agonists have an impact on mood and anxiety.

**Figure 1.**
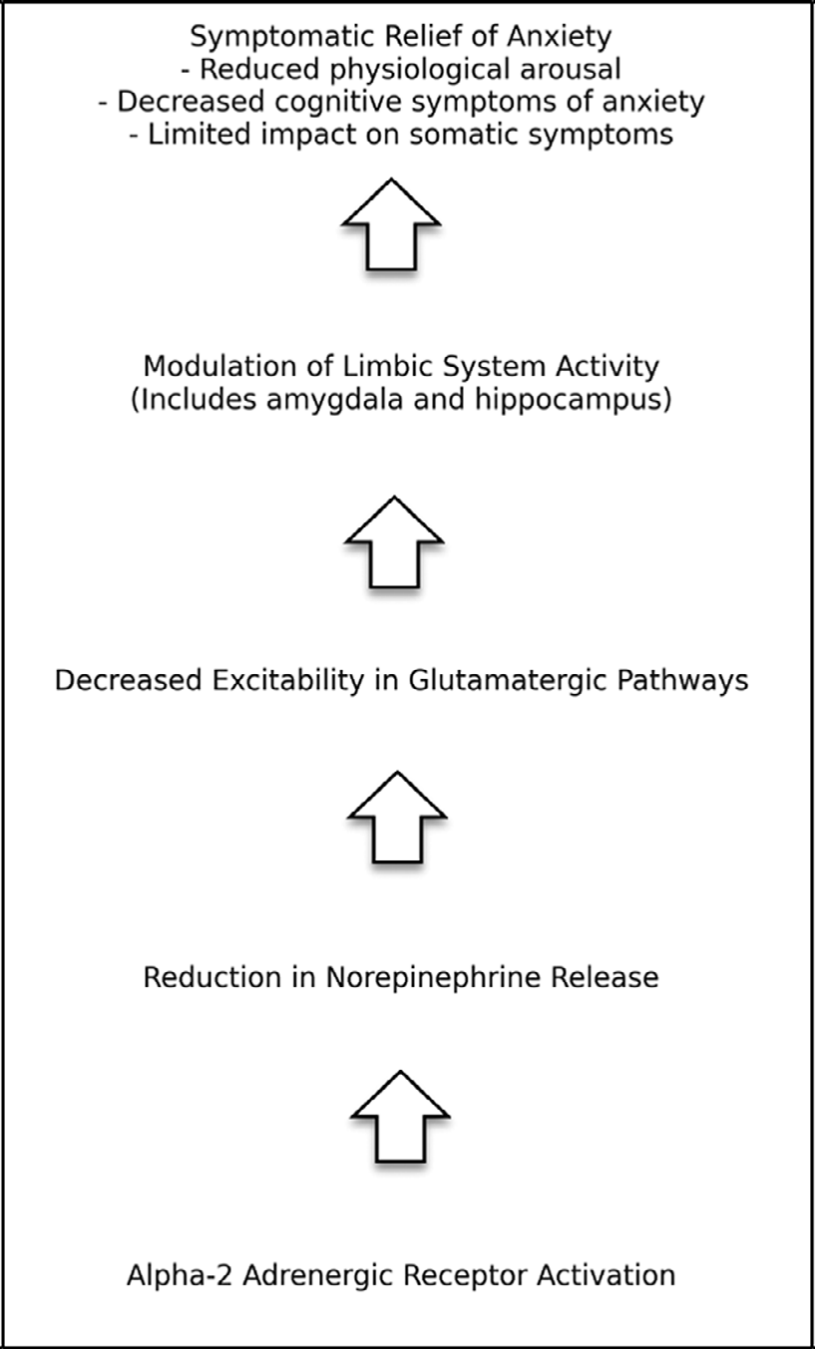
Mechanism of action of clonidine in anxiety disorders.

Clonidine works as an antihypertensive by lowering sympathetic flow to the brain and peripheral blood vessels.^12^ This also results in lower cardiac output and total peripheral resistance, which demonstrates its use as an antihypertensive.^13^ It is a fast-acting antihypertensive medication with twice-a-day oral dosing.^12^ However, in addition to its use in lowering blood pressure, clonidine also has some traditional off-label uses for managing other psychiatric disorders, including ADHD, post-traumatic stress disorder (PTSD), and Tourette Syndrome. Additionally, clonidine’s effects on postsynaptic receptors might contribute to its anxiolytic properties, although this dual action could explain the variability in its efficacy among different patients. Specifically, clonidine affects postsynaptic alpha-2A adrenergic receptors located in specific brain areas, including the prefrontal cortex and locus coeruleus. When these postsynaptic receptors are activated, they can inhibit adenylate cyclase, which lowers cyclic AMP (cAMP) levels. Therefore, potassium channels open and calcium channels close. These changes lead to hyperpolarization in the postsynaptic neuron, making the neuron less likely to fire, perhaps resulting in less phenotypic anxiety (Figure 1).

**Table 1.** Summary of Clinical Evidence on Clonidine for Anxiety Disorders

Study	Population	Design	Findings	Limitations
Hoehn-Saric et al. (1981)	GAD patients	Comparative observational	Reduced somatic symptoms of anxiety	Small sample size, inconsistent efficacy
Bromfalk et al. (2023)	Pediatric patients (post-op anxiety)	Double-blind randomized trial	Clonidine reduced postoperative anxiety compared to midazolam	Focused on acute pediatric cases
Sarangi et al. (2021)	16-year-old with OCD and anxiety	Case report	Clonidine monotherapy reduced symptoms compared to sertraline	Single case, no generalizability
Baldwin et al. (2005)	Adults with GAD	Clinical practice guidelines	Clonidine as potential treatment for anxiety disorders but not first-line	Lack of large-scale randomized trials
Abelson et al. (2006)	GAD patients	Clinical observational with comparative design	Reduced growth hormone response, blunted noradrenergic activity	Correlation, not causation established

*Abbreviations:* GAD, generalized anxiety disorder; PD, panic disorder; OCD, obsessive-compulsive disorder; PTSD, post-traumatic stress disorder; RCT, randomized controlled trial.

## Potential side effects

The use of clonidine in anxiety disorders has a side effect profile that is important to keep in mind. The reported side effects from clonidine include sedation, hypotension, and potential worsening of symptoms in some patients.^14^ These adverse effects indicate that careful monitoring and dosage adjustments may be necessary when prescribing it as a treatment. After abrupt cessation, clonidine was reported to have rebound hypertensive effects. Additionally, the heterogeneous nature of anxiety disorders implies that clonidine may not be universally effective for every individual, so its role may be more complementary to use in anxiety disorders rather than as the primary treatment option as there are not many studies investigating well-defined GAD, PD, SAD, PTSD, and obsessive-compulsive disorder (OCD).

## Evidence from clinical research

In a study by Hoehn-Saric et al., clonidine was evaluated in patients with GAD as well as PD using a double-blind crossover design method.^14^ The results suggested that clonidine significantly reduced anxiety attacks and “psychic” symptoms compared to placebo, although somatic symptoms were notably less affected.^14^ These research results indicate that clonidine may be particularly effective for the cognitive aspects of anxiety rather than physical symptoms experienced by patients. To assess its efficacy, clonidine has been compared with other more popular anxiolytic treatments, such as BZs and certain antidepressants. It was found that while clonidine can be effective, the benefits from the medication may not be as prevalent as those seen with more traditional anxiolytics like BZs, especially when it comes to the rapid symptom relief of anxiety-associated symptoms offered by BZs in particular.^14^ However, long-term studies suggest that while tolerance to clonidine’s sedative effects can develop, its anxiolytic properties can persist.^14^ This makes clonidine a viable option for patients who need medication for use over extended time periods and wish to experience less sedation, motor impairment, or addiction.

A double-blind randomized trial on 90 children, evaluated clonidine’s effects on postoperative anxiety in children compared to midazolam and intranasal dexmedetomidine.^13^ When evaluating whether postoperative anxiety was reduced, both clonidine and dexmedetomidine significantly reduced anxiety compared to midazolam.^15^ This suggests clonidine’s potential unique role in managing acute anxiety specifically in pediatric cases. This supports clonidine’s anxiolytic properties and indicates its effectiveness in decreasing anxiety in specific settings which can be taken into account when providers choose the best treatment options.

Another study demonstrated clonidine’s beneficial use in patients who have concurrent psychiatric diagnoses with anxious distress. A case report by Sarangi et al. presented a case of a 16-year-old female with OCD with comorbid PTSD, anxiety, and depression.^16^ Because initial treatment with SSRIs was not satisfactory for the patient, subsequent transdermal clonidine was tried due to its autonomic nervous system dampening effects.^16^ Clonidine monotherapy was found to significantly reduce anxiety symptoms associated with OCD and PTSD, demonstrating its potential benefits for treating anxiety symptoms across various psychiatric disorders.^16^ Similarly, a study by Hoehn-Saric et al. also assessed clonidine’s effects on individuals with PD.^14^ It was observed that clonidine could attenuate panic attacks, which aligns with the hypothesis that the drug’s modulation of the noradrenergic system can reduce the physical surges of anxiety characteristics that overlap with those seen in panic attacks.^14^ It is still important to note that the response to clonidine was inconsistent, with some patients experiencing worsening symptoms, highlighting the complexity of its usage and the need for individualized treatment approaches to best manage care. This research demonstrates the possible benefits of clonidine usage for cases with concurrent psychiatric diagnoses.

Several other clinical studies have investigated the efficacy of clonidine in anxiety disorders. Abelson et al. reported reduced growth hormone (GH) responses to clonidine in patients with GAD, suggesting a blunted noradrenergic response compared to healthy controls.^17^ Sullivan et al. observed exaggerated blood pressure responses to clonidine in PD patients, indicating altered autonomic reactivity.^18^ Furthermore, Schittecatte et al. noted diminished GH and REM sleep-suppressing effects of clonidine in depressed patients, highlighting potential differential responses based on comorbidities.^7^

Comparative studies have also explored clonidine’s efficacy relative to other medications. Baldwin et al. compared clonidine to SSRIs in the treatment of GAD and found comparable efficacy, suggesting clonidine as a viable alternative in certain patient populations.^19^ Clonidine has less weight gain and sexual side effects perhaps allowing it to be better tolerated than SSRI/SNRIs in some patients.

In conclusion from these comparative studies, clonidine’s pharmacological profile and clinical evidence support its potential benefits in treating anxiety disorders, particularly in cases of noradrenergic dysfunction or treatment-resistant symptoms though large-scale studies comparable to SSRIs/SNRIs and BZs do not yet exist. Further research, including large-scale RCTs, is warranted to elucidate its optimal dosing, safety profile, and long-term efficacy. Clonidine represents a possible promising avenue for expanding the therapeutic armamentarium for anxiety disorders, offering a novel mechanism of action, tolerability, and the potential for improved patient outcomes (Table 1).

## Clinical applications and integrative care

From a real-world clinical perspective, clonidine may be particularly useful in integrative psychiatry, where it could be combined with behavioral therapies, biofeedback, or lifestyle interventions to enhance anxiety management. Given its favorable side-effect profile compared to BZs and potential for off-label use in anxiety, further research should explore its role within holistic treatment paradigms. Future directions should examine how clonidine interacts with complementary treatments such as mindfulness-based interventions, acupuncture, or dietary modifications aimed at regulating the autonomic nervous system.

## Conclusion

Anxiety disorders represent a significant public health concern, affecting millions worldwide and contributing to substantial socioeconomic burdens. While existing treatments such as SSRI, SNRI, BZs, and CBT are effective for many patients, a considerable proportion experience treatment resistance or incomplete symptom relief. Additionally, the side effects of SSRI, SNRI, and BZs may preclude their use. This has prompted exploration into alternative pharmacotherapies, including the use of clonidine, an alpha-2 adrenergic receptor agonist with demonstrated effects on noradrenergic and glutaminergic pathways. Clonidine shows promise as an anxiolytic agent. Some studies and case reports suggest potential benefits, particularly in specific populations or when other treatments have failed. However, the current evidence is insufficient to recommend clonidine as a first-line treatment for anxiety disorders. More robust, large-scale studies are essential to establish clonidine’s role, optimal dosing, and safety profile in the treatment of anxiety disorders.

## Data Availability

All data supporting the findings of this study are included within the article and its supplementary files.
